# Body temperature measurement in mice during acute illness: implantable temperature transponder versus surface infrared thermometry

**DOI:** 10.1038/s41598-018-22020-6

**Published:** 2018-02-23

**Authors:** Jie Mei, Nico Riedel, Ulrike Grittner, Matthias Endres, Stefanie Banneke, Julius Valentin Emmrich

**Affiliations:** 1 Department of Neurology and Department of Experimental Neurology, Charité – Universitätsmedizin Berlin, corporate member of Freie Universität Berlin, Humboldt Universität zu Berlin, and Berlin Institute of Health, Berlin, Germany; 2 QUEST – Center for Transforming Biomedical Research, Berlin Institute of Health (BIH), Berlin, Germany; 3 Center for Stroke Research, Charité - Universitätsmedizin Berlin, Berlin, Germany; 4 Department of Biostatistics and Clinical Epidemiology, Charité - Universitätsmedizin Berlin, Berlin, Germany; 5 German Center for Neurodegenerative Diseases (DZNE), Berlin, Germany; 6 German Center for Cardiovascular Research (DZHK), Berlin, Germany; 7 German Federal Institute for Risk Assessment, German Center for the Protection of Laboratory Animals (Bf3R), Berlin, Germany

## Abstract

Body temperature is a valuable parameter in determining the wellbeing of laboratory animals. However, using body temperature to refine humane endpoints during acute illness generally lacks comprehensiveness and exposes to inter-observer bias. Here we compared two methods to assess body temperature in mice, namely implanted radio frequency identification (RFID) temperature transponders (method 1) to non-contact infrared thermometry (method 2) in 435 mice for up to 7 days during normothermia and lipopolysaccharide (LPS) endotoxin-induced hypothermia. There was excellent agreement between core and surface temperature as determined by method 1 and 2, respectively, whereas the intra- and inter-subject variation was higher for method 2. Nevertheless, using machine learning algorithms to determine temperature-based endpoints both methods had excellent accuracy in predicting death as an outcome event. Therefore, less expensive and cumbersome non-contact infrared thermometry can serve as a reliable alternative for implantable transponder-based systems for hypothermic responses, although requiring standardization between experimenters.

## Introduction

The Three Rs (Replacement, Reduction and Refinement) were introduced almost 60 years ago as guiding principles for humane animal research^1^. Since then, the application of humane end points, which allow early termination of experiments before animals experience significant harm while intended to ensure robustness and reproducibility of results, has become widely accepted. In mice, the most commonly used laboratory animals, body temperature is a fundamental parameter in the evaluation of animal wellbeing^2–5^. It is therefore important in mouse models of acute illness to be able to reliably assess temperature. However, to date, only a minority of mouse studies using endotoxin or infectious agents to elicit acute illness use temperature to monitor disease progression and define humane endpoints.

Traditionally, the mainstay of temperature monitoring in mice have been invasive measurement techniques such as rectal, tympanic or oesophageal probing and bladder or pulmonary artery catheterization allowing for measurement of core body temperature^6,7^. Of those, rectal thermometry is the most widely used means of temperature measurement in mice^8,9^. However, limitations of this procedure are its time-consuming application and distress to animals, which may cause an increase in core temperature, leading to a misinterpretation of the animal’s physiological state^10,11^. In addition, rectal probing can lead to mucosal tearing or infection^7,8^. In contrast, non-invasive temperature monitoring techniques such as non-contact infrared thermometry reduce animal discomfort and lower the risk for injury or cross contamination^12^. Common sites for surface temperature measurement in mice include the tympanic membrane, the back, sternum, abdominal region and ano-genital region, all of which correlate with core body temperature^3,8,13–17^. More recently, subcutaneous implantable passive radio-frequency identification (RFID) or active telemetry transponder systems for core temperature probing have become a reliable alternative and are widely considered best practice^6,8,11^. However, in spite of its usability, the high startup expenses, required surgical skills and distress to animals caused by the implantation procedure limit the widespread application of such systems.

The aim of this study therefore was to compare and assess the respective merits of two distinct methods to assess body temperature in mice during acute illness, namely the above implantable RFID transponder and non-contact infrared thermometry allowing for measurement of body core and surface temperature, respectively. To this end, we used a well-established endotoxin (lipopolysaccharide, LPS) model, which induces acute sickness behaviour over a period of around 36 hours accompanied by hypothermia. To allow for meaningful comparison, a subgroup of the same animals was used in either method. Furthermore, to encompass the entire severity spectrum of endotoxin-induced hypothermia, we used a large number of animals (n = 435) and four different mouse strains, namely C57BL/6 J and homozygous knockout strains for *Mfge8*, *Mertk*, and *Cd11b* (deficient for the phagocytic opsonin MFG-E8, the phagocytic receptor MerTK, and one subunit of the complement receptor 3, respectively), which demonstrate varying susceptibility to LPS. In addition, to assess both methods during normothermia, we studied saline-treated controls and animals with long recovery after LPS application for up to 7 days. To maximize generalizability of results, we used two different brands of standard non-contact infrared thermometers obtained at a local pharmacy to measure the animals’ surface temperature. To compare the two methods, we performed correlation analyses between core and surface temperature and used a mixed effects model to evaluate the surface temperature as a predictor of core temperature. Furthermore, we trained machine learning models with the temperature data obtained by the two methods to determine appropriate temperature thresholds and time points to be used as humane endpoints in this model. We then compared the accuracy of both methods in the prediction of death as an outcome event.

## Results

### Temperature change over time in LPS-treated and saline-treated animals

**Figure 1:**
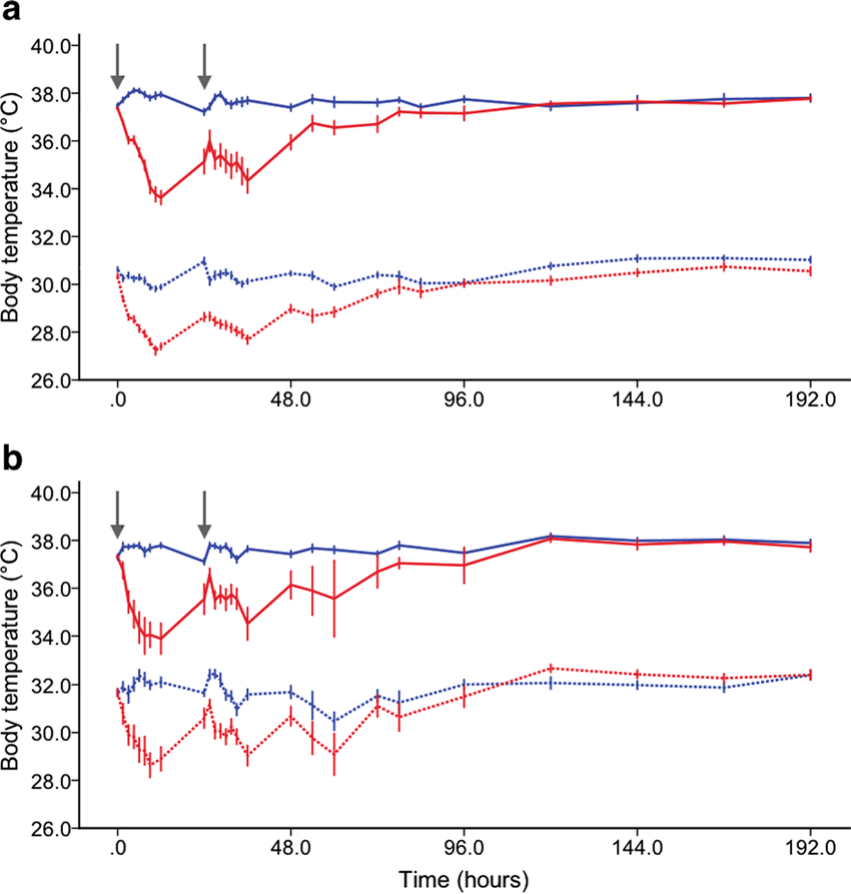
Line graphs showing body core and surface temperature across lipopolysaccharide (LPS)- and saline-treated groups for up to 7 days post-treatment. (**a**) Temperature profile obtained by implantable radio frequency identification (RFID) transponders and infrared thermometer 2 (n = 380; LPS-treated, 251; saline-treated, 129). (**b**) Temperature profile obtained by implantable RFID transponders and infrared thermometer 1 (n = 55; LPS-treated, 33; saline-treated, 22.). Blue, saline-treated control animals; red, LPS-treated animals; solid line, core temperature; dotted line, surface temperature; grey arrow, time of LPS/saline injections. Data shown are means ± 95% CI.

Figure 1 shows the mean (95% CI) body core and surface temperature by measurement modality across both LPS-treated and saline-treated groups. As expected, LPS-treated animals showed a pronounced decrease in both surface and core temperature on both injection days. The surface temperature of LPS-treated animals measured with infrared thermometer 1 reached a minimum at 9 hours following the first injection (28.6 (1.7) °C) and at 12 hours following the second injection (29.0 (2.1) °C). Core temperature of these animals was the lowest at 12 hours following the first (33.9 (2.7) °C) and second injection (34.5 (2.9) °C), respectively. The surface temperature of LPS-treated animals measured with infrared thermometer 2 was the lowest at 10.5 hours following the first injection (27.2 (1.8) °C) and 12 hours following the second injection (27.7 (2.4) °C). Core temperature of these animals reached the lowest value at 12 hours following the first (33.6 (2.4) °C) and second injection (34.3 (3.9) °C), respectively. Both core and surface temperature returned to baseline within 96 hours following the second LPS injection. Conversely, saline-treated animals showed a mild increase in both core and surface temperatures from baseline on both injection days, which can be attributed to handling stress caused by the injection and repetitive temperature measurements. Core temperature of saline-treated animals reached its daily maximum at 4.5 hours (38.0 (0.4) °C and 37.9 (0.5) °C) post-injection on both injection days. Surface temperature of animals measured with infrared thermometer 1 peaked at 6 hours following the first injection (32.4 (1.2) °C) and at 3 hours following the second injection (32.5 (0.8) °C), respectively. No significant increase in surface temperature from baseline was observed in saline-treated animals measured with infrared thermometer 2 (for details see Fig. 1 and Supplementary Table S1).

**Figure 2:**
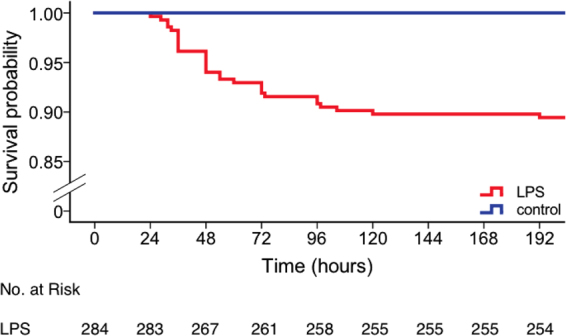
Kaplan-Meier curve showing cumulative survival across lipopolysaccharide (LPS)- and saline-treated animals for up to 7 days post-injection. Blue, saline-treated control mice (n = 151, n(dead) = 0); red, LPS-treated mice (n = 284, n(dead) = 30).

Strain- or genotype- dependent effects on the core and surface temperatures were observed among LPS-treated animals (for details see Figure S1). Following the first injection, homozygous knockout and control animals showed a similar hypothermic response to LPS for core and surface temperatures alike. However, following the second injection, the hypothermic response to LPS was markedly less severe in Cd11b knockout and control animals (35.6 (0.8) °C, 35.1 (2.5) °C, 31.7 (6.0) °C and 31.9 (6.0) for the lowest core temperature on injection day 2 for homozygous Cd11b knockout, pooled homozygous wildtype controls, Mertk knockout, and Mfge8 knockout animals, respectively). There was no significant genotype-dependent effect on core temperature (p = 0.9, F = 0.18, repeated measures ANOVA). In contrast, we observed a genotype-dependent effect on surface temperature (p < 0.001, F = 10.2). Interestingly, following the second injection, Cd11b knockout animals had a significantly reduced surface temperature until the end of the experiment when compared to controls (p < 0.001, repeated measures ANOVA). Thirty out of 284 LPS-treated animals (89%) survived for up to 7 days after the first injection (Fig. 2). Among the 30 dead animals, 18 were found dead and 12 were euthanized after reaching predetermined humane endpoint criteria, illustrating a distinct weakness of the sickness behaviour score which was used for this study to identify an animal’s distress (Table 1, Supplementary Table S3). Death occurred from 24 to 192 hours after the first injection (average = 60.5 (35.1) h). Table 1 shows the mean (SD) last recorded temperature before death and time of death per strain. All saline-treated animals survived.

**Table 1:** Number of dead and transponder-implanted animals, time of death and last recorded core and surface temperatures before death following lipopolysaccharide (LPS) injection per genotype. No transponder-implanted Cd11b animals died, thus their near-death core temperature was not assessed. N/A: not applicable.

	C57BL/6	Mertk	Cd11b	Mfge8
Number of dead animals	3 (1 euthanized, 2 found dead)	17 (4 euthanized, 13 found dead)	2 (2 euthanized)	8 (5 euthanized, 3 found dead)
Number of dead animals with transponder implant	3	4	0	8
Core temperature before death (°C)	max = 28.7 min = 24.9 average = 26.6 (1.6)	max = 29.3 min = 22.3 average = 24.2 (3.0)	N/A	max = 31.6 min = 20.5 average = 24.5 (3.7)
Surface temperature before death (°C)	max = 25.9 min = 24.4 average = 25.1 (0.5)	max = 28.9 min = 20.2 average = 23.3 (1.9)	max = 21.3 min = 19.0 average = 20.3 (0.9)	max = 27.1 min = 21.2 average = 23.4 (1.9)
Time of death (hours after the first injection)	max = 104.5 min = 36.0 average = 66.8 (30.1)	max = 192.0 min = 31.5 average = 67.6 (39.9)	max = 72.0 min = 54.0 average = 63.0 (9.9)	max = 97.5 min = 24.0 average = 42.6 (22.8)

### Comparison of measurement modalities

Surface temperature of the heating pad measured with infrared thermometer 1 was 1.7 °C higher than surface temperature measured with infrared thermometer 2 (infrared thermometer 1: 37.5 (2.2) °C; infrared thermometer 2: 35.8 (0.9) °C), indicating different calibration settings. Surface temperature across all animals was 5.7 °C (core temperature, 36.9 (1.9) °C; surface temperature, 31.2 (1.8) °C) and 6.9 °C (core temperature, 36.3 (2.5) °C; surface temperature, 29.4 (2.1) °C) lower than core temperature for infrared thermometer 1 and 2, respectively (Fig. 1).

Temperature acquisition by implantable RFID transponders was hampered by technical issues such as transponder readout error, dislodged and lost transponders, and reader failure leading to partial or full exclusion of 31, 14, and 6 out of a total of 199 transponder-implanted animals, respectively. In case of readout error or reader failure, available data from before and/or after the technical problem was used in the analysis, which was defined as a partial exclusion of experimental data. Animals with dislodged and lost transponders were entirely excluded from further analysis. During surface temperature acquisition, an unexpected technical error by an untrained experimenter led to falsely increased readings in 13 Mfge8 mice on injection day 2. At 10.5 hours following the first injection core and surface temperature values were not recorded in animals measured with infrared thermometer 1 because of human error.

### Correlation and prediction of core from surface temperature

**Figure 3:**
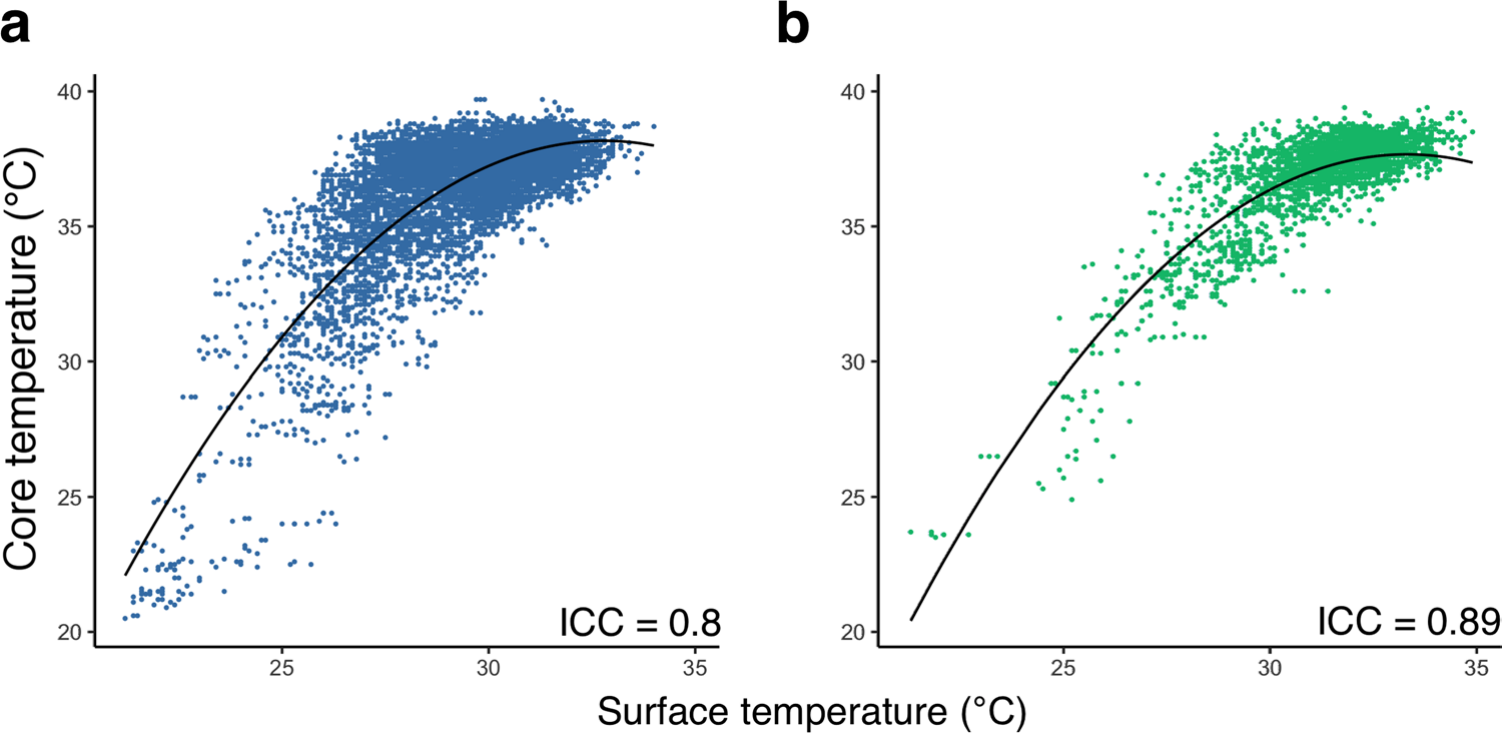
Prediction of core temperature from surface temperature using a mixed effects model. (**a**) Surface temperature measured by thermometer 2, plotted against the corresponding core temperature (n = 124, ICC = 0.80 (95%CI: 0.79–0.81)) and (**b**) surface temperature measured by thermometer model 1, plotted against the corresponding core temperature (n = 53, ICC = 0.89 (95%CI: 0.88–0.90)) show a positive non-linear correlation. A fitted mixed effects model was used to predict the corresponding core temperature from surface temperature. Marginal R2 (R2m): 0.65, conditional R2 (R2c): 0.81; Black solid line, fit line of the mixed effects model showing the core temperature predicted from surface temperatur.        ${\rm{Fit}}\,{\rm{line}}\,{\rm{in}}\,({\rm{a}}),y=-0.12\times {(x-30)}^{2}+0.92\times (x-30)+35.48$        ${\rm{fit}}\,{\rm{line}}\,{\rm{in}}\,({\rm{b}}),y=-0.12\times {(x-30)}^{2}+0.8\times (x-30)+36.36$

There was high inter-method consistency between surface and core body temperature across all animals and treatment groups (intra-class correlation coefficient, ICC = 0.89; 95%CI: 0.88–0.90; n = 53 and 0.80; 95%CI: 0.79–0.81; n = 124 for infrared thermometer 1 and 2, respectively; Fig. 3).

A positive non-linear relationship was found between core and surface temperatures regardless of the infrared thermometer being used, indicating that the rate of increase in core temperature slowed with higher surface temperatures. The nonlinear relationship between surface and core temperature was strong at low temperatures thus allowing for predictions of core temperature from surface temperature under hypothermic conditions. However, the relationship was weaker at normothermia due to variation of the surface temperature.

Relationship between the core and surface temperature could be described as follows:

If core temperature is predicted from measures by infrared thermometer 1:

1 $$\begin{array}{rcl}core\_temperature & = & -0.12\times {(surface\_temperature-30)}^{2}+\\  &  & 0.8\times (surface\_temperature-30)+36.36\end{array}$$

If core temperature is predicted from measures by infrared thermometer 2:

2 $$\begin{array}{rcl}core\_temperature & = & -0.12\times {(surface\_temperature-30)}^{2}\\  &  & +0.92\times (surface\_temperature-30)+35.48\end{array}$$

In this study, the marginal R^2^ (R2m) was 0.65 and the conditional R^2^ (R2c) was 0.81, indicating a good model fit.

### Using core temperature or surface temperature to predict death

To compare the accuracy of both core and surface temperature in the prediction of death as an outcome event, we developed a temperature-based death prediction model using machine learning algorithms. To understand whether models trained by core or surface temperature could achieve comparable performance in death prediction, a two-step approach was applied. First, core and surface temperatures from transponder-implanted animals were taken, and used separately to train the prediction models (n = 160). Since surface temperature of only 3 dead mice was measured with infrared thermometer 1, death prediction was not conducted separately for each infrared thermometer. After a comprehensive parameter space search, comparison of core- and surface-temperature-based prediction models was conducted. This was followed by selection of the best performing models, which were trained with data from all animals whose surface temperature measurements were available at 36 hours after the first injection (n = 372; for details see Supplementary Table S2).

A parameter search with models including support vector machine, logistic regression and random forest classifier showed that an F1 score of >0.9 could only be achieved when temperature data of up to 120 hours was available (Supplementary Table S2). With surface temperature data from 36 hours after the first injection, the precision and F1 scores dropped by 0.06 and 0.08 when switching from a more complex support vector machine model to a decision tree model, respectively (n = 160). However, the differences in precision and F1 scores dropped to 0 and 0.03 when data from additional animals was used (n = 372). The mean prediction accuracy reduced by 0.01 when using a decision tree model instead of a support vector machine model for both sets of animals (n = 160 and n = 372, respectively; Supplementary Table S2). Therefore, a decision tree model of depth 1 was used for its general performance and low complexity. Animals that were not assessed 36 hours after the first injection were not included in the analysis. All models were tested with multiple combinations of temperature values from different post-injection time points (for details see Supplementary Table S2).

Death could be predicted with high accuracy both from core and surface temperature (accuracy = 96.3%, F1 = 0.77 and accuracy = 95.6%, F1 = 0.69 for core and surface temperatures, respectively; n = 160, number of dead animals = 13). Surface temperature data from 372 mice (number of dead animals: 28) led to an accuracy of 96.5% and a F1 score of 0.76. Accordingly, there was excellent agreement between core and surface temperature to predict death as an outcome event.

Using the above model, application of a temperature threshold of 28.1 °C or 24.3 °C (for core or surface temperature, respectively) at 36 hours after the first LPS-injection would have allowed for early termination of experiments for 13 out of 19 animals at this time point, thus avoiding otherwise unnecessary suffering and distress.

## Discussion

The aim of this study was to compare and assess the respective merits of two commonly used procedures to assess body temperature in mice during acute illness following an endotoxin challenge. Two main strengths of this study are the large number of animals used and that the two methods were evaluated within the same animals allowing for an optimal comparison.

**Figure 4:**
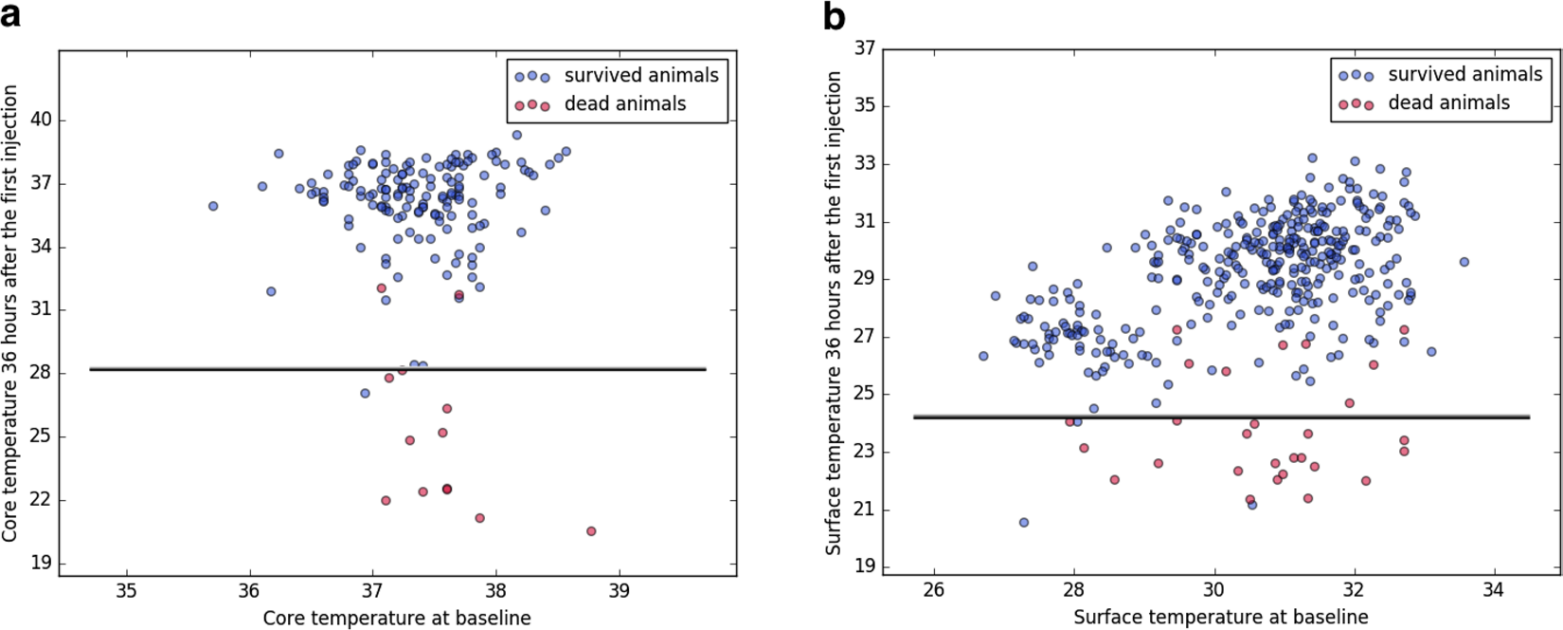
Predicting death using threshold models trained with core or surface temperature. (**a**) Core temperature (n = 160, number of dead animals = 13) or (**b**) surface temperature (n = 372, number of dead animals = 28) at 36 hours after the first injection was plotted against baseline temperature values. Blue and red dots represent measurements of survived and dead animals, which were used in the training, testing and validation of the prediction model, with one dot indicating measurements of one animal. Black solid lines indicate the decision boundaries determined by the prediction model trained with core or surface temperatures. If an animal’s body temperature falls into the area below the decision boundary (i.e., core temperature <28.1 °C or surface temperature <24.3 °C), the animal is predicted to die at a later time point. Applying a combination of the two thresholds would have allowed for early termination of experiments before animals experienced further distress for 13 out of 19 animals, which died at later time points during the study. Applying thresholds individually would have resulted in early termination of experiments for 4 out of 7 transponder-implanted animals or 12 out of 19 animals with surface temperature recordings, respectively.

To summarize our results, both methods produced similar findings in mice during normothermia and following LPS-induced hypothermia and the inter-method consistency was high (Figs 1 and 3). As expected and depicted in Fig. 1, surface temperature was considerably lower than core temperature throughout the experiment. Surface temperature measured by infrared thermometer 1 was higher than that of infrared thermometer 2 despite similar core temperature values indicating that the difference in surface temperature between the two infrared thermometers was most likely caused by different default calibration settings. In addition, the variance of surface temperature measurements was about twice as high than that of core temperature (Supplementary Table S1). However, using a mixed model approach, core temperature could be predicted reliably from surface temperature and death as an outcome event could be predicted with high accuracy and precision based on surface and core temperatures alike using machine learning algorithms (Figs 3 and 4). It would therefore appear from the present study that although surface temperature measurements have higher degrees of variation and are less sensitive to subtle changes in temperature, this method is well suited to determine temperature-based humane endpoint criteria.

Previous studies have addressed the correlation between core temperature and surface temperature. Among studies involving core and surface temperature measurements in mice, the surface temperature is on average 2.57 °C, 3–4 °C or 2–3 °C lower than core temperature, depending on the site of measurement and restraints applied (sternum, back without restraints and back with restraints, respectively) and there is moderate to strong correlation between core and surface temperatures during hypothermia^3,8,13^. An important factor contributing to differences in absolute temperature between an animal’s core and surface is ambient temperature. The lower the ambient temperature, the lower the surface temperature, whereas the core temperature stays constant as long as thermoregulatory responses are intact^18,19^. Thus, variations between studies can at least partly be attributed to variations in ambient temperature. Furthermore, we and others have shown that surface temperature has poor predictive value for core temperature at normothermia^7,20–22^ (Fig. 3). This is most likely caused by changes in skin vasomotricity, which occur in phase with physiological thermoregulatory events, i.e. regular waves of vasoconstriction and vasodilatation of vessels in the skin in areas such as the paws and tail^23^. It is likely that the perineal area, which is close to the base of the tail and which was used for temperature measurement in this study, would be affected in the same way. In contrast, surface temperature has good predictive value in hypothermia and hyperthermia when thermoregulatory responses are insufficient to maintain core temperature ^6,13,17,23^. Another factor contributing to differences between core and surface temperature is handling stress. Restraining devices were used in previous studies for probe-based surface temperature acquisition, however, body temperature may start to increase within seconds of a mouse being restrained^3,8,10,24^. Stress results in activation of the sympathetic nervous system, which in turn leads to increased thermogenesis and vasoconstriction of skin vessels resulting in divergence of core and surface temperature^25–27^. In the present study, the perianal region was used for its accessibility and need for only minimal animal handling during surface temperature acquisition. Thus, the difference between core and surface temperatures can be attributed to variations in ambient temperature, measurement location and thermoregulatory responses, handling stress, and the intentional use of standard non-contact infrared thermometers without a calibration feature. Nevertheless, we found a strong correlation between core and surface temperatures.

Genotype-dependent effects on body temperature were most pronounced in homozygous Cd11b knockout animals whose hypothermic response to LPS was significantly reduced following the second injection, which might be attributable to a conditioning effect of repeated inflammatory stimuli. In addition, Cd11b knockout animals had a lower surface temperature following the second LPS injection which might be due to an altered thermoregulatory response of this genotype.

A threshold determined by body temperature was used as a refined humane endpoint in previous studies. However, the threshold for determining the risk of death was selected based on a less exhaustive approach: for example, plotting the mortality against body temperature, using receiver operating characteristic (ROC) curves to evaluate the relationship between lowest recorded temperature versus survival, analysis of sensitivity and specificity of death prediction of certain cut-off values, or/and a selection based on average/lowest temperatures in treated versus untreated animals^2,3,9,13,15^. These methods only allow a parameter search with large increments (0.5–1 °C) in the predictor (i.e., body temperature) in a less systematic manner, thus compromising the accuracy of prediction. To identify the parameter that could be used as the humane endpoint in the present study, we applied an automatized parameter search with finer increments in the predictor (0.01–0.1 °C) to approximate the threshold criteria. In addition, previous studies refrained from conducting a comprehensive assessment of prediction models (e.g., random forests, decision tree, logistic regression and support vector machine) or parameters (e.g., average surface/core temperature and/or lowest surface/core temperature at individual time points or across several time points, respectively) and their various combinations for death prediction. To our knowledge, this is the first study to determine a temperature-based threshold and to compare the prediction accuracies of core vs. surface temperature using machine learning algorithms in a mouse model of acute disease.

Factors other than variation of measurements need to be considered when assessing the relative merits of these two methods. Regarding surface temperature measurements, a standard non-contact infrared thermometer and only minimal training and technical expertise are sufficient. In contrast, core temperature measurements using temperature transponders require a dedicated RFID system consisting of passive (usually non-reusable) RFID transponders and a reader device; transponder implantation needs to be performed by an experimenter with previous surgical experience followed by regular checks for transponder functionality and dislodgement. With respect to time efficiency, non-contact infrared thermometry allows to obtain a measurement in 3–4 seconds, compared to 10–30 seconds for scanning and obtaining a measurement, including a re-calibration time, using the RFID reader system as used here. Thus, in the hands of a skilled animal technician, using infrared thermometry is likely to reduce handling time and animal distress. However, surface temperature measurements are prone to higher inter- and intra-subject variation and highly investigator-dependent, whereas transponder-based readings are more robust and less investigator-dependent. Table 2 summarizes the advantages and drawbacks of the two methods.

**Table 2:** Advantages and limitations of the two temperature measurement techniques assessed in the present study.

	Implantable temperature transponder	Non-contact infrared thermometer
Required equipment	Expensive	Standard
Required preparation	Transponder Implantation	None
Regular checks for functionality	Required for transponder readers and transponders	Required for thermometers
Variation of measurements	Optimal	Good
Time efficiency/Time of animal handling	Poor (~10–30 seconds per measurement)	Good (~3–4 seconds per measurement)
Level of technical expertise required	High	Standard
Experimenter-dependency	Standard	High
Accuracy to predict death as an outcome event	High	High

One important conclusion from our study is therefore that, given the above constraints of using implantable RFID temperature transponders, infrared thermometry is acceptable as surrogate whenever variation of measurements can be counterbalanced with multiple measurements or large numbers of animals. However, to quantify subtle changes in temperature requires the use of the former despite being more cumbersome, expensive, and time consuming. As the result, both core and surface temperature are equally suited to predict death allowing for termination of experiments at earlier time points to reduce unnecessary distress.

Limitations of our study include the use of only one disease model, namely LPS-induced hypothermia. We chose an endotoxin model for its reproducibility and high translational relevance, as hypothermia is a common feature during severe illness in mice^28–30^. It would nevertheless be of interest to compare surface and core temperature measurements during hyperthermia as produced by stress or pharmacological intervention. Another limitation is the proximity of the RFID transponder’s implantation site to brown adipose tissue located in the interscapular region. Brown adipose tissue is an important heat generator in mice and thus might have a confounding effect on core temperature readings^31^. Only female mice were used for this study. However, the stage of the oestrous cycle was not determined. Thus, physiological temperature fluctuations with the oestrous cycle may have had a confounding effect on temperature measurements. Both core and surface temperature gave rise to comparable accuracy in death prediction. However, due to the small number of animals that died in the present study (6.9%), survived and dead cases were severely imbalanced, which may lead to an inflated accuracy estimate in death prediction. Therefore, we calculated precision scores and F1 scores for models with high accuracy estimates to examine their performance in a more comprehensive manner, which showed excellent agreement between the two methods (Supplementary Table S2). Finally, because of transponder malfunction and technical errors during temperature acquisition, we had to exclude core temperature measurements from 22 animals partially or in their entirety from further analysis.

In conclusion, this is the first study to apply systematic assessment of two distinct methods of temperature measurement in mice following an endotoxin challenge and to compare their predictive strengths towards death as an outcome event. We find that both methods are adequately suited for the prediction of death, and hence that the less expensive and cumbersome non-contact infrared thermometry can serve as a reliable alternative for implantable transponder-based systems. This finding is of practical importance as it encourages adoption of simple temperature measurement tools to monitor disease progression and apply humane endpoints in mouse models of acute disease.

## Materials and Methods

All experimental protocols were approved by the Landesamt für Gesundheit und Soziales, Berlin (Reg 239/15) and were conducted in accordance with the German animal protection law and local animal welfare guidelines. Reporting of the study complies with the ARRIVE (Animal Research: Reporting of *In Vivo* Experiments) guidelines^32^.

### Animals, housing and husbandry

Female, 2 months old C57BL/6 J mice were derived from Charles River. *Mertk* (Jax: B6;129-Mertk tm1Grl/J), *Cd11b* (Jax: B6;129-Mertk tm1Grl/J, B6.129S4-Itgam tm1Myd/J, and *Mfge8*^33^ (provided by C. Théry, INSERM 932, France) knockout mice were derived from The Jackson Laboratory and Hertie Institute for Clinical Brain Research, respectively, and bred locally. Female homozygous knockout mice and their homozygous wildtype littermates were used in experiments at the age of 8–10 weeks (total: n = 435. C57BL/6 J: n = 55; Mertk: n = 126; Cd11b: n = 126; Mfge8: n = 128. Animals were kept in specific-pathogen-free (SPF) conditions according to FELASA regulations and group-housed with *ad libitum* access to food and water in type III polycarbonate cages equipped with environmental enrichment tools (red transparent plastic nest box and brown paper towels). During acute illness and recovery, animals were housed individually in custom-made polycarbonate cages (20 × 20 × 30 cm) from 48 h before the first injection until 72 h after the second injection, after which they were returned to their home group cage. Room temperature was maintained at 23.0 ± 1.0 °C with a relative humidity between 55 and 65%. Animals were kept under a 12:12 h light:dark cycle (lights on: 20.00, lights off: 8.00) and were exposed to white noise at moderate intensity (65 dB) during the dark phase (Dohm Sleepmate, Marpac Sound Machines, Wilmington, USA). To minimize confounding effects, injections and temperature measurements were scheduled at the same time each day and experimenters wore single-use coveralls (Microgard 1500, Ansell Microgard, Kingston Upon Hull, UK), gloves and surgical masks whenever in contact with animals.

### Methods to prevent bias

Animals were randomized for treatment, measurement modality, and survival times using the Research Randomizer tool (https://www.randomizer.org) by a researcher who was not involved in the injection procedure or temperature measurements. Information on strain and treatment group assignment was concealed from experimenters until the end of the study.

### Exclusion criteria and humane endpoints

There were no specific exclusion criteria. A scoring system based on general activity and response to stimuli was adapted from the murine sepsis score as shown in Supplementary Table S3 to determine disease progression and humane endpoint criteria^34^. Severity of disease was scored on a scale from 0 to 5 (normal score, 0; maximum severity: 5). When the general activity and response to stimuli of an animal matched criteria from different severity levels, the average of the two severity levels was assigned as the sickness score. Upon reaching a score larger than 4 once or a score of 4 twice within 2 hours, animals were immediately removed from the cage and killed by cervical dislocation. On the two consecutive injection days, animals were scored eight times daily (8:00 to 20:00, every 90 min). On recovery days 2 and 3 after the first injection, three times daily (8:00 to 20:00, every 6 h) and once a day (8:00) from post-injection day 3 until the end of the experiment.

### Transponder implantation, anaesthesia, and temperature measurement

**Figure 5:**
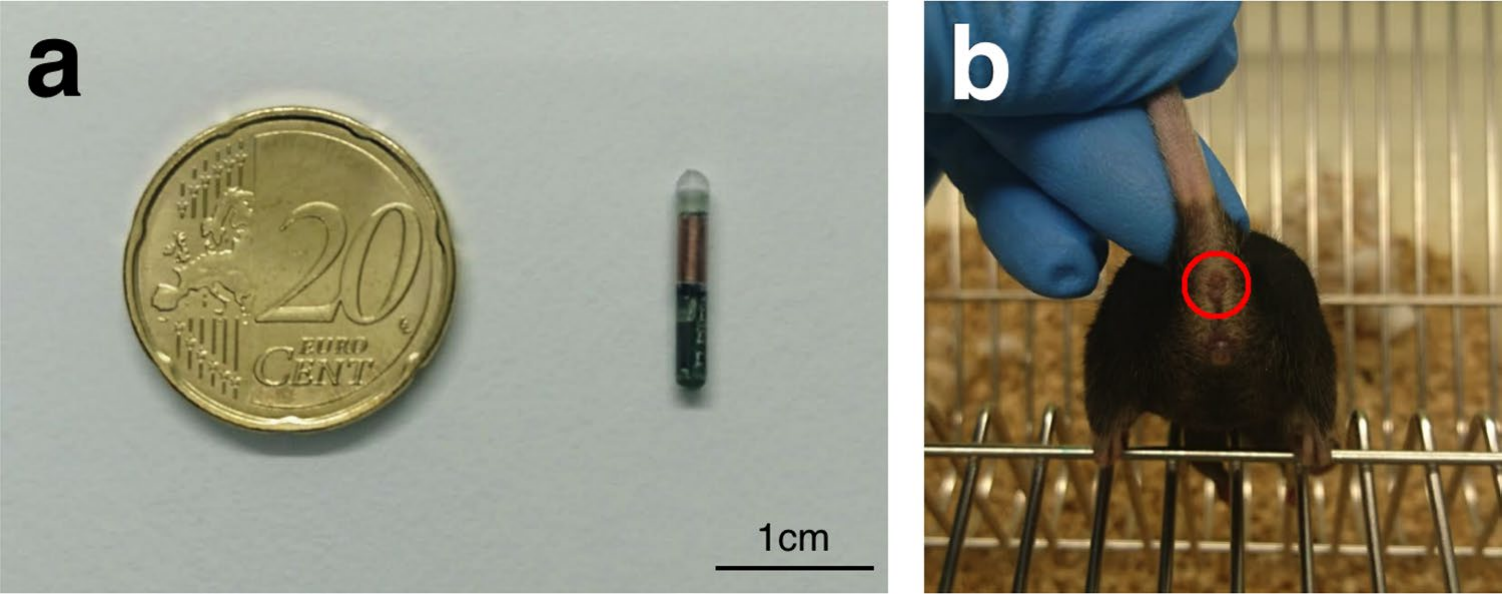
Illustration of the measurement modalities used to obtain core and surface temperature data. (**a**) Size comparison of a temperature transponder to a 20-cent coin. (**b**) Mouse with the ano-genital area exposed. Red circle depicts perianal region used as the site for surface temperature acquisition.

For temperature acquisition using radio-frequency identification (RFID) technology, passive RFID transponders were implanted subcutaneously. When the passive RFID transponder is within read range, its internal antenna draws energy from the radio waves emitted by the reader. This energy powers the chip, which then sends data back to the reader. Before implantation, the glass-covered, biocompatible temperature transponders (dimension: 2 mm × 14 mm; model: IPTT-300 transponders; BioMedic Data Systems, Seaford, USA) were programmed with individual identification numbers, loaded in a needle applicator device, and sterilized (Fig. 5a). Three weeks prior to injection, temperature transponders were implanted subcutaneously in the region between the scapulae as described previously^13,35^. Anaesthesia was induced with 2% isoflurane delivered in 100% oxygen for <45 s before the implantation procedure and injected once with meloxicam (1 mg/kg; Sigma-Aldrich, USA) for analgesia. Following implantation, mice were observed for up to 48 hours for signs of complications and temperature transponders were checked weekly for presence and functionality before the start of the experiment.

A non-contact handheld transponder reader (DAS-7008/9; BioMedic Data Systems, Seaford, USA) was used to read the implanted transponder. During temperature acquisition, the animal was placed on the experimenter’s palm with the tail gently fixed. The head of the handheld reader was held 2–3 cm above the animal’s shoulder region, with a slow circular movement until the temperature reading was displayed on the reader.

### Non-contact infrared thermometry and temperature measurement

Non-contact infrared thermometers measure the infrared energy emitted by an object for estimating its temperature and can only be used to monitor surface temperatures. In this study, two non-contact infrared thermometer models (model 1: Braun No touch – NTF3000; Braun, Kronberg, Germany; model 2: Aponorm Contact Free 3; WEPA Aponorm, Hillscheid, Germany) were obtained from a local pharmacy. For direct comparison, both thermometer models were initially used to measure the temperature of a heating pad (Hot Plate 062; Labotect, Göttingen, Germany) over a temperature range from 31 to 39 °C with an increment of 0.5 °C. During subsequent temperature acquisition, the base of the tail was fixed with two fingers and then gently lifted while the animal gripped a metal rod on the cage lid with its front paws, thus allowing for exposure of the ano-genital area. Temperature was measured in the perianal region, with a searchlight indicating the measured area (Fig. 5b). To minimize confounding effects by urination or defecation, temperature measurements were only taken when the measurement area was clean. Infrared temperature measurements were taken over a readout time of approximately 3–4 seconds with the thermometer held 1–2 cm from the reading site.

### Timeline of temperature monitoring

Baseline body temperature readings were obtained at 8 am at the day of the first injection. On the two consecutive injection days body temperature was obtained eight times daily (8:00 to 20:00, every 90 min), three times daily (8:00 to 20:00, every 6 h) on recovery days 2 and 3 after the first injection, and once a day (8:00) from post-injection day 3 until the end of the experiment (for details see Supplementary Figure S2). The sequence of core or surface temperature measurements was randomized. Handling was minimized to reduce stress and discomfort.

### Endotoxin-induced systemic inflammatory response

To induce a systemic inflammatory response, animals were treated with lipopolysaccharide (LPS), a cell wall component of Gram-negative bacteria. LPS (from Salmonella enterica serotype, Sigma-Aldrich St. Louis, USA) at a dose of 1.5 mg/kg or physiological phosphate-buffered saline solution were administered intraperitoneally on two consecutive days at the beginning of the active (i.e. light-off) phase at 8:00 with a volume of 10 μl/g.

### Experimental design

Experimental animals were divided into 5 groups with different survival times: 3 hours (n = 44: 16 Mertk, 14 Cd11b and 14 Mfge8), 1 day (n = 63: 10 C57BL/6, 19 Mertk, 17 Cd11b and 17 Mfge8), 3 days (n = 76: 10 C57BL/6, 18 Mertk, 25 Cd11b and 23 Mfge8), 7 days (n = 66: 11 C57BL/6, 16 Mertk, 19 Cd11b and 20 Mfge8) and 60 days (n = 186: 24 C57BL/6, 57 Mertk, 51 Cd11b and 54 Mfge8), respectively. Survival times were determined to fulfil the objectives of another study, for which these animals were used. Infrared thermometer 1 was used in the assessment of surface temperature in C57BL/6 mice (n = 55). Infrared thermometer 2 was used for temperature acquisition in Mertk (n = 126), Cd11b (n = 126), and Mfge8 (n = 128) mice.

### Data analysis and statistics

For all animals, temperature measurements were repeated in triplicate. Results are expressed as mean (SD) unless otherwise specified. Data processing and statistical analysis was performed using SPSS version 24 (SPSS Inc., Chicago, IL, USA), R 3.3.3 (R Development Core Team) and Python 2.7.10 (Python Software Foundation, https://www.python.org). Intra-class correlation coefficients (ICC) and 95% confidence intervals (CI) were used to analyse the level of agreement between surface and core temperature measures. To assess the reliability of surface temperature in predicting the corresponding core temperature, a random intercept mixed effects model^36,37^ with 3 levels was used to fit the data (1st level, temperature measures; 2nd level, time points where measures from different temperature monitoring methods were combined; 3rd level, animals), for its advantage in dealing with missing values caused by different survival times and measurement intervals. Risk of death as an outcome event was examined with the scikit-learn toolkit (sklearn)^38^ for both core and surface temperature. Core and surface temperature were analysed separately with (1) temperature data from 12 and 36 hours after the first injection; (2) lowest temperature by 24 hours for the first 48 hours post-injection (i.e., the lowest temperatures for hours 0–24 and for hours 24–48); and (3) average temperature by 24 hours for the first 48 hours post-injection (i.e., the average temperatures for hours 0–24 and for hours 24–48). The three parameter sets were used individually or in combination with logistic regression model, decision tree model, support vector machine, and random forest classifier with a 3-fold (n = 160) or 5-fold (n = 372) stratified cross-validation.

### Data availability

Three datasets including (1) surface, core temperature and sickness score, (2) other information (treatment group assignment, strain/genotype and survival status) of all animals and (3) temperature readings from a heating pad obtained with the two thermometer models are available as open data on Figshare Repository in raw data format: Figs 1, 3 and 4 and Table 1 (Dataset 1), 10.6084/m9.figshare.5589883; Fig. 2 and Table 1 (Dataset 2), 10.6084/m9.figshare.5589892, temperature readings from a heating pad obtained with the two thermometer models (Dataset 3), 10.6084/m9.figshare.5765991.

## Electronic supplementary material

Supplementary Materials
