# Supplementary material for: Body temperature measurement in mice during acute illness: implantable temperature transponder versus surface infrared thermometry
Source: Sci Rep. 2018 Feb 23;8:3526. doi: 10.1038/s41598-018-22020-6 (PMC5824949; doi:10.1038/s41598-018-22020-6)
Supplement: Supplementary file 1 — Supplementary Materials [file 41598_2018_22020_MOESM1_ESM.pdf]

Supplementary information for:

Body temperature measurement in mice during acute illness: implantable temperature transponder versus surface infrared thermometry

AUTHORS

Jie Mei[1], Nico Riedel[2], Ulrike Grittner[3,4], Matthias Endres[1,3,5,6], Stefanie Banneke[7], Julius Valentin Emmrich[1]*

AFFILIATIONS

1. Department of Neurology and Department of Experimental Neurology, Charité – Universitätsmedizin Berlin, corporate member of Freie Universität Berlin, Humboldt Universität zu Berlin, and Berlin Institute of Health
2. QUEST – Center for Transforming Biomedical Research, Berlin Institute of Health (BIH)
3. Center for Stroke Research, Charité - Universitätsmedizin Berlin
4. Department of Biostatistics and Clinical Epidemiology, Charité - Universitätsmedizin Berlin
5. German Center for Neurodegenerative Diseases (DZNE), Berlin, Germany
6. German Center for Cardiovascular Research (DZHK), Berlin, Germany
7. German Federal Institute for Risk Assessment, German Center for the Protection of Laboratory Animals (Bf3R),Berlin, Germany

* corresponding author: Julius V. Emmrich, Department of Neurology and Department of Experimental Neurology, Charitéplatz 1, Berlin 10117, Germany. Email: julius.emmrich@charite.de

Figures

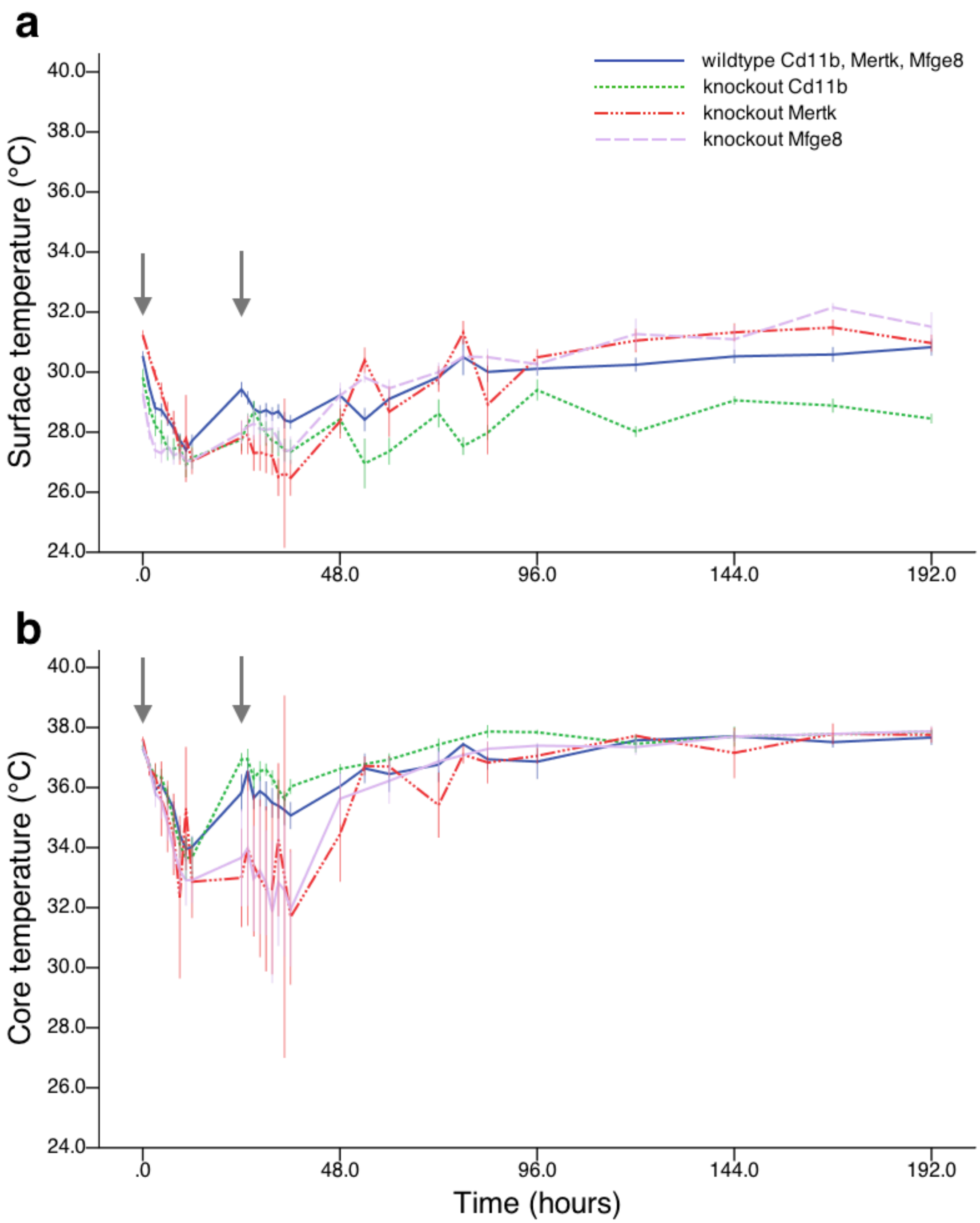

Figure S1. Line graphs showing body core and surface temperatures by genotype following LPS injection. For clarity, temperature data of control homozygous wildtype Mertk, Cd11b and Mfge8 animals was pooled. (a) Temperature profile obtained by infrared thermometer 2 (surface temperature). (b) Temperature profile obtained by implantable RFID transponders (core temperature). Grey arrow, time of LPS injections. Data shown are means (95% CI).

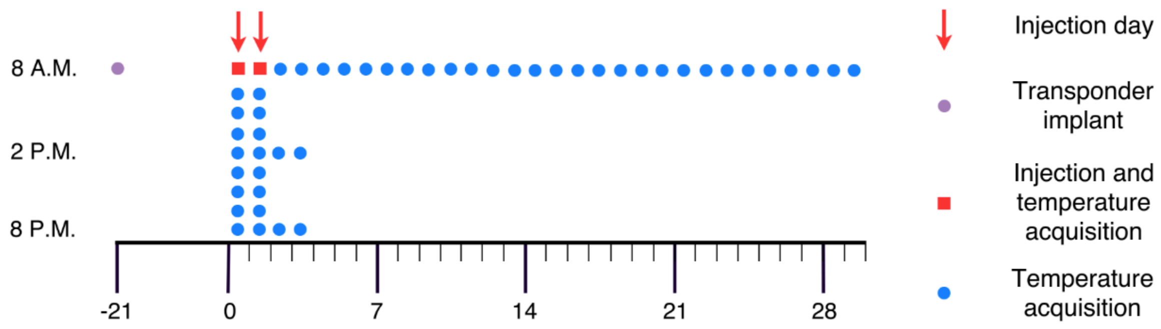

Figure S2. Experimental timeline. Animals were implanted with temperature transponders 21 days prior to the first lipopolysaccharide (LPS) or saline injection. Baseline body temperature readings were obtained at 8.00 before the first injection. LPS or saline was injected on two consecutive days followed by a recovery period of up to 7 days.

Tables

Table S1. Mean body temperature (°C) at selected time points during the temperature monitoring period. Animals were injected with saline (control) or lipopolysaccharide (LPS) on two consecutive days and their core or surface temperature was assessed with temperature transponders or one of two non-contact infrared thermometers (infrared thermometer 1 and 2, respectively); t, time (hours) since the first LPS injection; N/A, not available.

			t = 0	t = 6	t = 9	t = 10.5	t = 12	t = 24	t = 36	t = 48	t = 72	t = 96	t = 120
Control	Animals measured with infrared thermometer 1	Core temperature	max = 38.5<br>min = 36.2<br>average = 37.4<br>SD = 0.5<br>n = 22	max = 38.9<br>min = 36.6<br>average = 37.9<br>SD = 0.5<br>n = 20	max = 38.6<br>min = 36.7<br>average = 37.8<br>SD = 0.5<br>n = 14	max = N/A<br>min = N/A<br>average = N/A<br>SD = N/A<br>n = N/A	max = 39.0<br>min = 37.1<br>average = 37.9<br>SD = 0.5<br>n = 19	max = 38.6<br>min = 36.1<br>average = 37.2<br>SD = 0.5<br>n = 22	max = 38.5<br>min = 36.7<br>average = 37.7<br>SD = 0.5<br>n = 19	max = 38.6<br>min = 36.2<br>average = 37.5<br>SD = 0.6<br>n = 21	max = 38.4<br>min = 36.3<br>average = 37.5<br>SD = 0.5<br>n = 19	max = 38.2<br>min = 36.6<br>average = 37.5<br>SD = 0.4<br>n = 18	max = 39.2<br>min = 37.2<br>average = 38.3<br>SD = 0.5<br>n = 16
		Surface temperature	max = 33.7<br>min = 30.0<br>average = 31.7<br>SD = 0.9<br>n = 22	max = 34.7<br>min = 29.6<br>average = 32.4<br>SD = 1.2<br>n = 20	max = 33.7<br>min = 30.8<br>average = 32.0<br>SD = 0.8<br>n = 14	max = N/A<br>min = N/A<br>average = N/A<br>SD = N/A<br>n = N/A	max = 34.3<br>min = 29.3<br>average = 32.1<br>SD = 1.0<br>n = 19	max = 33.1<br>min = 29.8<br>average = 31.7<br>SD = 0.8<br>n = 22	max = 33.5<br>min = 28.2<br>average = 31.6<br>SD = 1.1<br>n=19	max = 34.2<br>min = 28.0<br>average = 31.7<br>SD = 1.3<br>n = 21	max = 33.9<br>min = 28.7<br>average = 31.5<br>SD = 1.2<br>n = 19	max = 33.9<br>min = 30.5<br>average = 32.0<br>SD = 0.9<br>n = 18	max = 34.4<br>min = 28.7<br>average = 32.1<br>SD = 1.1<br>n = 16
	Animals measured with infrared thermometer 2	Core temperature	max = 38.6<br>min = 36.5<br>average = 37.5<br>SD = 0.5<br>n = 30	max = 39.2<br>min = 36.5<br>average = 38.1<br>SD = 0.4<br>n = 30	max = 38.5<br>min = 36.1<br>average = 37.8<br>SD = 0.5<br>n = 26	max = 38.8<br>min = 36.3<br>average = 37.9<br>SD = 0.5<br>n = 19	max = 39.0<br>min = 36.3<br>average = 37.9<br>SD = 0.5<br>n = 29	max = 38.6<br>min = 36.2<br>average = 37.2<br>SD = 0.6<br>n = 24	max = 39.3<br>min = 36.3<br>average = 37.7<br>SD = 0.7<br>n = 27	max = 38.6<br>min = 34.9<br>average = 37.4<br>SD = 0.7<br>n = 31	max = 39.1<br>min = 36.6<br>average = 37.6<br>SD = 0.7<br>n = 23	max = 38.7<br>min = 36.5<br>average = 37.7<br>SD = 0.6<br>n = 23	max = 38.5<br>min = 36.8<br>average = 37.4<br>SD = 0.5<br>n = 11
		Surface temperature	max = 33.2<br>min = 27.0<br>average = 30.6<br>SD = 1.1<br>n = 128	max = 32.8<br>min = 27.0<br>average = 30.3<br>SD = 1.1<br>n = 79	max = 32.5<br>min = 27.2<br>average = 29.9<br>SD = 1.0<br>n = 88	max = 32.9<br>min = 27.8<br>average = 29.8<br>SD = 1<br>n = 74	max = 32.5<br>min = 26.0<br>average = 29.9<br>SD = 1.1<br>n = 113	max = 36.5<br>min = 25.8<br>average = 31.0<br>SD = 1.6<br>n = 104	max = 32.9<br>min = 27.1<br>average = 30.1<br>SD = 1.1<br>n = 109	max = 33.1<br>min = 27.3<br>average = 30.5<br>SD = 1.1<br>n = 114	max = 32.9<br>min = 26.5<br>average = 30.4<br>SD = 1.2<br>n = 101	max = 32.9<br>min = 23.9<br>average = 30.1<br>SD = 1.5<br>n = 101	max = 32.9<br>min = 27.6<br>average = 30.8<br>SD = 1.2<br>n = 83

Table S1 continued.

			t = 0	t = 6	t = 9	t = 10.5	t = 12	t = 24	t = 36	t = 48	t = 72	t = 120
LPS	Animals measured with infrared thermometer 1	Core temperature	max = 38.4<br>min = 36.5<br>average = 37.4<br>SD = 0.4<br>n = 31	max = 38.4<br>min = 26.4<br>average = 34.4<br>SD = 2.6<br>n = 21	max = 37.2<br>min = 32.2<br>average = 34.1<br>SD = 1.6<br>n = 12	max = N/A<br>min = N/A<br>average = N/A<br>SD = N/A<br>n = N/A	max = 38.7<br>min = 30.3<br>average = 33.9<br>SD = 2.7<br>n = 23	max = 37.9<br>min = 23.6<br>average = 35.6<br>SD = 3.1<br>n = 31	max = 37.8<br>min = 24.9<br>average = 34.5<br>SD = 2.9<br>n = 24	max = 38.0<br>min = 26.5<br>average = 36.2<br>SD = 2.6<br>n = 26	max = 38.3<br>min = 23.5<br>average = 36.7<br>SD = 2.9<br>n = 23	max = 39.1<br>min = 37.0<br>average = 38.2<br>SD = 0.5<br>n = 15
		Surface temperature	max = 33.8<br>min = 28.6<br>average = 31.7<br>SD = 1.1<br>n = 33	max = 33.9<br>min = 24.9<br>average = 29.3<br>SD = 2.3<br>n = 23	max = 32.8<br>min = 26<br>average = 28.6<br>SD = 1.7<br>n = 13	max = N/A<br>min = N/A<br>average = N/A<br>SD = N/A<br>n = N/A	max = 34.0<br>min = 25.2<br>average = 28.9<br>SD = 2.4<br>n = 25	max = 33.7<br>min = 21.8<br>average = 30.6<br>SD = 2.2<br>n = 33	max = 33.2<br>min = 24.4<br>average = 29.0<br>SD = 2.1<br>n = 26	max = 33.7<br>min = 23.0<br>average = 30.7<br>SD = 2.0<br>n = 28	max = 33.5<br>min = 21.3<br>average = 31.1<br>SD = 2.2<br>n = 25	max = 34.3<br>min = 31.1<br>average = 32.7<br>SD = 0.8<br>n = 17
	Animals measured with infrared thermometer 2	Core temperature	max = 38.9<br>min = 35.7<br>average = 37.4<br>SD = 0.5<br>n = 90	max = 38.7<br>min = 31.1<br>average = 35.5<br>SD = 1.6<br>n = 86	max = 38.4<br>min = 26.6<br>average = 34.1<br>SD = 2.0<br>n = 69	max = 38.8<br>min = 28.7<br>average = 33.8<br>SD = 2.1<br>n = 54	max = 38.6<br>min = 24.3<br>average = 33.6<br>SD = 2.4<br>n = 82	max = 38.4<br>min = 21.1<br>average = 35.1<br>SD = 3.8<br>n = 66	max = 38.9<br>min = 20.5<br>average = 34.3<br>SD = 3.9<br>n = 76	max = 38.2<br>min = 22.5<br>average = 35.9<br>SD = 2.5<br>n = 76	max = 38.6<br>min = 23.0<br>average = 36.7<br>SD = 2.3<br>n = 55	max = 38.7<br>min = 36.5<br>average = 37.5<br>SD = 0.5<br>n = 27
		Surface temperature	max = 33.4<br>min = 26.3<br>average = 30.3<br>SD = 1.6<br>n = 251	max = 32.1<br>min = 21.5<br>average = 28.1<br>SD = 2.0<br>n = 200	max = 33.7<br>min = 22.5<br>average = 27.6<br>SD = 1.9<br>n = 177	max = 31.8<br>min = 22.6<br>average = 27.2<br>SD = 1.8<br>n = 109	max = 32.9<br>min = 22.4<br>average = 27.4<br>SD = 2.0<br>n = 214	max = 32.9<br>min = 21.4<br>average = 28.6<br>SD = 2.3<br>n = 189	max = 32.1<br>min = 20.3<br>average = 27.7<br>SD = 2.4<br>n = 191	max = 33.2<br>min =19.3<br>average = 29.0<br>SD = 2.3<br>n = 203	max = 33.6<br>min = 21.0<br>average = 29.6<br>SD = 2.1<br>n = 163	max = 33.2<br>min = 26.9<br>average = 30.2<br>SD = 1.8<br>n = 118

Table S2. Performance of machine learning models for death prediction in some of the tested parameter/model combinations. Scores of one of the best models were listed for each parameter combination. Performance of the decision tree with input data obtained at 36 hours after the first injection was included in comparison with the best-performing model. 1: temperature at the 12th and 36th hour after the first injection; 2: lowest temperature during 48 hours following first injection; 3: average temperature during 48 hours following the first injection; 4: lowest temperature during 120 hours following the first injection; 5: average temperature during 120 hours following the first injection; 6: temperature at the 36th hour after the first injection; SVC, support vector machine; rbf, radial basis function.

Model	Averaged accuracy	Averaged precision	F1 score	Parameter	Number of Animals	Site of temperature assessment
SVC rbf, C=100, gamma=0.0001	0.99	0.89	0.93	1, 4, 5	152	core
SVC rbf, C=10, gamma=0.0001	0.99	1	0.95	1, 4, 5	152	surface
SVC rbf, C=1, gamma=0.001	0.99	0.925	0.94	1, 4, 5	359	surface
Logistic Regression	0.98	0.92	0.87	1, 2, 3	152	core
Random Forest, n_estimators = 4	0.96	1	0.75	1, 2, 3	152	surface
SVC rbf, C=1, gamma=0.01	0.97	0.9	0.79	1, 2, 3	359	surface
SVC rbf, C=1, gamma=0.1	0.98	0.92	0.87	1	152	core
SVC rbf, C=10, gamma=0.01	0.97	0.89	0.75	1	152	surface
SVC rbf, C=100, gamma=0.001	0.97	0.9	0.79	1	359	surface
SVC rbf, C=1, gamma=0.01	0.98	0.92	0.88	6	160	core
Decision Tree, max_depth=1	0.96	0.78	0.77	6	160	core
SVC linear, C=1	0.97	0.89	0.77	6	160	surface
Decision Tree, max_depth=1	0.96	0.83	0.69	6	160	surface
SVC rbf, C=100, gamma=0.01	0.97	0.89	0.79	6	372	surface
Decision Tree, max_depth=1	0.96	0.89	0.76	6	372	surface

Table S3. Scoring criteria for the severity of sickness by general activity and response to stimuli as adapted from Shrum *et al*[34].

Score	Definition
**0**	• Mouse is any of: eating, drinking, climbing, running, and fighting<br>• No observable changes in spontaneous movements<br>• Mouse responds immediately to auditory stimulus or touch
**1**	• Slightly suppressed activity<br>• Moderate response to auditory stimulus<br>• Strong response to touch (moves to escape)
**2**	• Moderately suppressed activity<br>• Weak response to auditory stimulus<br>• Strong response to touch (moves to escape)
**3**	• Severely suppressed activity<br>• Mouse is stationary with occasional investigative movements<br>• No response to auditory stimulus<br>• Moderate response to touch (moves a few steps)
**4**	• Severely suppressed activity<br>• Mouse moves only when provoked<br>• No response to auditory stimulus<br>• Mild response to touch (moves a few steps)
**5**	• No spontaneous activity<br>• Mouse remains stationary when provoked<br>• Mouse experiencing tremors<br>• No response to auditory stimulus<br>• Little or no response to touch (no locomotion)<br>• Cannot right itself if pushed over
